# Towards an ecosystem of clinical decision support tools for precision cancer medicine

**DOI:** 10.1038/s41698-026-01551-6

**Published:** 2026-06-17

**Authors:** Sigve Nakken, Anders Edsjö, Janne Lehtiö, Vigdis Nygaard, Paul Roepman, Richard Rosenquist, Hege G. Russnes, Albrecht Stenzinger, David Tamborero, Sen Zhao, the PCM4EU consortium, Anders Edsjö, Albrecht Stenzinger, David Tamborero, Agnieszka Janowska, Alba López Rioja, Anni Lepland, Antonio Marra, Anu Ustav, Åslaug Helland, Attila Patocs, Beatrice Mainoli, Bettina Ryll, Birute Brasiuniene, Daniel Kazdal, Ebba Hallersjö Hult, Edita Baltruškevičienė, Eduard Vrdoljak, Egbert Smit, Eivind Hovig, Elena Chavarria, Elena Garralda, Eline Aas, Elisa Bjørgo, Giuseppe Curigliano, Gro Live Fagereng, Hans Gelderblom, Hans Timmer, Hege G. Russnes, Henk Verheul, Henriett Butz, Irene Brana, Iwona Lugowska, Izabela Agnieszczak, Jean-Yves Blay, Julio Oliveira, Kadri Rekker, Kadri Toome, Katriina Jalkanen, Kjetil Taskén, Knut Smeland, Kristoffer Staal Rohrberg, Loic Verlingue, Martin Højgaard, Mika Mustonen, Paola Zagami, Ragnhild Sørum Falk, Rasa Sabaliauskaite, Richard Rosenquist, Rui Henrique, Sahar Barjesteh van Waalwijk van Doorn-Khosrovani, Sigmund Brabrand, Simon Ekman, Soemeya F. Haj Mohammad, Sonata Jarmalaite, Tanja Juslin, Tiina Kahre, Tina Kringelbach, Tormod Guren, Ulrik Lassen, Vigdis Nygaard, Vince Kornél Grolmusz, Eivind Hovig

**Affiliations:** 1 Department of Tumor Biology, Institute for Cancer Research, Oslo University Hospital, Oslo, Norway; 2 Centre for Cancer Cell Reprogramming, Institute of Clinical Medicine, University of Oslo, Oslo, Norway; 3 Department of Clinical Genetics, Pathology and Molecular Diagnostics, Skåne University Hospital, Lund, Sweden; 4 Department of Oncology-Pathology, Karolinska Institutet, Science for Life Laboratory, Stockholm, Sweden; 5 Department of Pathology, Division of Laboratory Medicine, Oslo University Hospital, Oslo, Norway; 6 Hartwig Medical Foundation, Amsterdam, the Netherlands; 7 Department of Molecular Medicine and Surgery, Karolinska Institutet, and Clinical Genetics and Genomics, Karolinska University Hospital, Stockholm, Sweden; 8 Institute for Cancer Research, Oslo University Hospital, Oslo, Norway; 9 Institute of Clinical Medicine, University of Oslo, Oslo, Norway; 10 Institute for Pathology, Heidelberg University Hospital, Heidelberg, Germany; 11 International Affairs Office, Maria Sklodowska-Curie Institute of Oncology, Warsaw, Poland; 12 Vall d’Hebron Institute of Oncology (VHIO), Vall d’Hebron Barcelona Hospital Campus, Barcelona, Spain; 13 Genetics and Personalized Medicine Clinic, Department of Personalized Medicine, Tartu University Hospital, University of Tartu, Estonian Cancer Network, Tartu, Estonia; 14 European Institute of Oncology IRCCS, Department of Oncology and Hemato-Oncology, University of Milano, Milano, Italy; 15 Oncology and Haematology Clinic, North Estonia Medical Centre, Institute of Clinical Medicine, University of Tartu, Tartu, Estonia; 16 Division of Cancer Medicine, Oslo University Hospital, Oslo, Norway; 17 Department of Molecular Genetics and the National Tumour Biology Laboratory, National Institute of Oncology, Budapest, Hungary; 18 Instituto Português de Oncologia do Porto FG, Porto, Portugal; 19 Stockholm School of Economics Institute for Research, Stockholm, Sweden; 20 Center of Medical Oncology, National Cancer Center of Vilnius University Hospital Santaros Clinics, Vilnius, Lithuania; 21 Faculty of Medicine, Vilnius University, Vilnius, Lithuania; 22 Institute of Pathology, University Hospital Heidelberg, Heidelberg, Germany; 23 Clinical Research Center, National Cancer Institute, Vilnius, Lithuania; 24 Department of Oncology, University Hospital Center Split, School of Medicine, University of Split, Split, Croatia; 25 Department of pulmonary diseases, Leiden University Medical Hospital, Leiden, The Netherlands; 26 Medical Oncology Service, Vall d’Hebron Barcelona Hospital Campus, Vall d’Hebron Institute of Oncology (VHIO), Barcelona, Spain; 27 Department of Health Management and Health Economics, University of Oslo, Division of Health Services, Norwegian Institute of Public Health, Oslo, Norway; 28 Division for Cancer Medicine, Oslo University Hospital, Oslo, Norway; 29 Department of Medical Oncology, Leiden University Medical Centre, Leiden, the Netherlands; 30 Department of Pathology and Institute for Cancer Research, Oslo University Hospital, Oslo, Norway; 31 Department of Medical Oncology, Erasmus MC Cancer Institute, University Medical Center Rotterdam, Rotterdam, the Netherlands; 32 Department of Molecular Genetics, National Institute of Oncology, Budapest, Hungary; 33 Center for Excellence in Precision Oncology, Maria Sklodowska-Curie National Institute of Oncology, Warsaw, Poland; 34 Centre Leon Berard, Lyon, France; 35 Department of Laboratory Genetics, Genetics and Personalized Medicine Clinic, Tartu University Hospital, Tartu, Estonia; 36 Comprehensive Cancer Center, Helsinki University Hospital, Helsinki, Finland; 37 Department of Oncology, Oslo University Hospital, Oslo, Norway; 38 Department of Oncology, Rigshospitalet, Copenhagen University Hospital, Copenhagen, Denmark; 39 Department of Research and Innovation, Centre Leon Berard, Lyon, France; 40 FICAN South, Southern Finland Cancer Center, Helsinki University Hospital and University of Helsinki, Helsinki, Finland; 41 Oslo Centre for Biostatistics and Epidemiology, Oslo University Hospital, Oslo, Norway; 42 National Cancer Institute, Vilnius, Lithuania; 43 Department of Molecular Medicine and Surgery, Karolinska Institutet, and Clinical Genetics, Karolinska University Hospital, Stockholm, Sweden; 44 Dept. of Medical Oncology, Leiden University Medical Centre, CZ Health Insurance, Leiden, the Netherlands; 45 Department of Oncology-Pathology, Karolinska Institutet, Karolinska University Hospital, Theme Cancer, Stockholm, Sweden; 46 Institute of Biosciences, Life Sciences Centre, Vilnius University, Vilnius, Lithuania; 47 Department of Laboratory Genetics, Genetics and Personalized Medicine Clinic, Tartu University Hospital, Institute of Clinical Medicine, University of Tartu, Tartu, Estonia; 48 Deptartment of Oncology, Rigshospitalet, Copenhagen University Hospital, Copenhagen, Denmark

**Keywords:** Cancer, Computational biology and bioinformatics

## Abstract

Molecular tumor boards are supported by clinical decision support system (CDSS) tools for the interpretation of complex molecular data towards personalized treatments. Current CDSS tools are genomics-focused and characterized by tight couplings between data ingestion, guideline-based reasoning logic and curated knowledgebases. Next-generation CDSS solutions should integrate multimodal data and learn from outcomes in an interoperable ecosystem, leveraging explainable, trustworthy output from AI models through rigorous clinical validation processes.

## Introduction

With the advent of high-throughput sequencing technologies, the potential of precision cancer medicine (PCM) has already shown significant clinical utility^1,2^. The array of detectable genomic alterations in a given tumor, paired with the increasing number of biomarker-guided drugs, effectively makes more cancer types treatable. As the number of possible interventions grows, selecting the most optimal treatment combination, based on the maximum amount of information at any given time point, becomes increasingly complex, both in clinical trials and individual experimental therapies. To address this complexity, the field utilizes sophisticated data interpretation tools for the identification and prioritization of evidence to support specific clinical actions. Such tools are broadly referred to as clinical decision support system (CDSS) tools.

What specifically defines a CDSS in the context of PCM efforts? We here define this as software solutions that can ingest and carefully prioritize patient-level molecular tumor data through the lens of curated biomarker knowledge and treatment guidelines, ultimately serving discussions in multidisciplinary molecular tumor boards (MTBs)^3^. Although recommendations and guidelines on the precise role and mission of MTBs have been proposed^4,5^, there is still significant institutional heterogeneity in MTB practices and operation^6^, a state that has created considerable variability with respect to the needs and expectations as to what a CDSS-like tool should provide. First, the boundaries between the output of high-throughput assay-dependent bioinformatics pipelines and the processed level of data ingested by the CDSS, are often not clear-cut. More importantly, however, is the unevenness of access and expertise regarding molecular diagnostics and high-throughput sequencing tests, which creates varied needs of the CDSS tools. While many MTB structures primarily focus on the detection of actionable genomic mutations from targeted gene panel assays, others aim to comprehensively interrogate whole-genome and other multi-omic profiling data for downstream treatment recommendations and trial inclusion^7^. Notably, MTB processes are also commonly framed by local constraints, such as specific evidence-level schemes, trial opportunities, and regulatory procedures for off-label drug use^8^, which influences how CDSS tools should identify and prioritize clinically actionable evidence^9^. Furthermore, as many centers are transitioning MTBs from special-case forums toward routine clinical practice, CDSS platforms are gradually expected to connect with local clinical IT workflows and seamlessly integrate data from conventional molecular pathology assays (morphology, imaging, immunohistochemistry, and fluorescence in situ hybridization). The heterogeneity and evolving nature of MTB operations in precision oncology thus illustrate a broad spectrum of CDSS requirements across hospitals and institutions, some of which are suboptimally fulfilled among the current generation of tools. Moreover, while efforts are ongoing to create core PCM data models to support harmonized clinical decision-making across healthcare systems, the adaptation of such standardized models into CDSS tools is still in its infancy^10^.

While previous reviews have emphasized tools and services that can support CDSS platforms for precision oncology^3,11^, our perspective proposes an architectural paradigm shift for next-generation solutions. Drawing on experiences with established genomics-focused CDSS frameworks developed within the academic community, we argue for a transition from monolithic and static reporting tools toward an adaptive ecosystem of modular services that can represent a more sustainable solution to meet the heterogeneous needs of MTBs. Interoperability is critical for the workings of such an ecosystem, and depends not only upon well-established community standards, but also on considerable computational resources and strong hospital IT infrastructure. In light of an increasing availability of data modalities for each cancer patient, we further argue how the emergence of generative AI represents a potentially paradigm-shifting development in such an ecosystem, particularly in the context of increasing data federation. Given the considerable impact CDSS tools may have on clinical outcomes, objective performance metrics and explainability will likely play a critical role in influencing the preferred strategy for implementing CDSS tools in PCM settings.

## Conventional CDSS tools for precision oncology

In PCM, CDSS solutions are currently heavily focused on genomic variant data interpretation, with the primary goal of translating the spectrum of molecular aberrations identified in a tumor sample towards evidence-based recommendations for biomarker-guided treatment. CDSS developed within the community are available as web portals, through application programming interfaces (APIs), and as stand-alone command-line tools^8,12–15^. These tools typically generate structured reports that often serve as key resources during MTB discussions. The technical backends constitute a mix of modular variant annotation workflows and rule-based software engines, integrating multiple gene and variant databases as well as different bioinformatics methods to prioritize variants for clinical and functional significance. Notably, annotated variants (e.g., single nucleotide variants (SNVs)/insertions-deletions (InDels), copy-number variants (CNV) and structural variants) make up the primary input data for most tools, reflecting the current strong enrichment of cancer biomarkers at the DNA level. While some CDSS also support the interpretation of RNA-based transcriptomics data (e.g., bulk gene expression and gene fusions), their integration with DNA findings remains limited.

Manually curated knowledge databases are essential resources for CDSS tools. Examples include OncoKB, CIViC, and CKB^16–18^, which enable the annotation of variants with functional or oncogenic impact. However, only a very limited set of variants have been thoroughly curated regarding their oncogenic effects, leaving the majority with an unknown classification. In this regard, rapidly emerging AI-based variant effect predictors (e.g., AlphaMissense and EVE) hold great promise as complements to manual curation procedures, although tools in this landscape are still considered research-grade^19,20^. Standard operating procedures to systematically collect and weigh oncogenicity have been established by ClinGen/CGC/VICC^21^. A clinical actionability assessment typically involves three steps. After an initial classification of variants for oncogenic or pathogenic impact, they are next matched for clinical relevance using external knowledgebases. These databases focus on evidence linking individual aberrations in particular tumor types to diagnostic classifications, prognostic outcomes, or drug sensitivity/resistance. The third operation involves a prioritization or tier classification of biomarker-matched aberrations by jointly considering the strength of evidence in the knowledgebases and the specific cancer type subject to interrogation. Importantly, the specific criteria or rules to assign variants to tiers of clinical actionability have been formalized in guidelines by professional societies such as AMP/ ASCO/CAP^22^ and ESMO^23^. The goal of such guidelines is to establish standardized communication of the clinical actionability of molecular findings. However, since CDSS tools vary in how they utilize and harmonize knowledge databases, and because different actionability recommendation frameworks co-exist across countries and continents, tools may produce discordant classifications or recommendations for the same variant^24^. This inconsistency, which directly impacts patient care, also highlights the inherent difficulty of comparing the performance and outputs of different CDSS tools within the field.

Features extending beyond the impact and actionability of single-gene alterations tend to be secondary within the current generation of tools. The analysis of complex biomarkers that capture broader genomic instability and the tumor immune landscape, e.g., homologous recombination deficiency, microsatellite instability (MSI), tumor mutational burden, HLA type, and mutational signatures (e.g., smoking or UV exposure), is still evolving when it comes to standards for measurement and tumor-specific interpretation^25,26^, with some (e.g., MSI) being considerably more mature than others. Concerning the utility of bulk RNA-seq data, so-called comparative transcriptomics is a dominating approach^27,28^, which aims to identify tumor gene expression outliers by comparing the gene expression levels in the input sample to the distributions in large-scale reference cohorts. This approach can help validate candidate targets identified at the DNA level and establish novel actionable targets. Gene expression clustering is also a powerful approach to identify cancer subtypes^29^. Finally, some tools do indeed annotate germline events and classify their clinical significance^8,14,15,30^. However, robust inference for the combined impact of cancer-predisposing germline variants and somatically acquired alterations, which can have important therapeutic implications in individual patients (e.g., biallelic BRCA loss and poly(ADP-ribose) polymerase (PARP) inhibition^31^), is often not fully supported and rather left to manual interrogation.

## Challenges with conventional CDSS tools

Although conventional CDSS solutions that support MTBs have laid the foundational framework for guideline-based actionability assessments of genomic aberrations, their performance and sustainability encounter challenges on multiple fronts. The quality, completeness, and timeliness of manually curated knowledge databases on drug-variant relationships remain to be one of the most critical features, where publicly available, open-source resources co-exist with licensed databases, typically curated using different conventions. Judging and understanding the quality of biomarker associations in multiple curated resources represents a continuous struggle for any CDSS effort, where the overarching goal is to incorporate emerging high-quality evidence in a timely manner, such as novel opportunities for off-label drug use or newly discovered mechanisms of drug resistance.

Moreover, the capacity to provide truly integrative and multimodal analyses is still in its infancy within current CDSS tools, although this can partly be attributed to the low clinical maturity of multi-omic profiling. Cross-omic data integration procedures do, however, carry considerable value for downstream interpretation. Notably, for putatively actionable DNA-level alterations, especially those that are commonly found at low allelic fractions in the tumor^32^, or for variants of uncertain significance more broadly, the integration of RNA-seq and proteomics data could provide a critical functional context. Furthermore, RNA-seq can confirm whether a critically mutated gene is actively transcribed, while proteomics can provide insights into whether the corresponding protein is expressed or post-translationally modified in ways that may affect its function^33^. In parallel, advanced profiling of the tumor microenvironment, enabled by computational pathology technologies or single-cell assays, will undoubtedly become increasingly accessible. Such multi-layered evidence has the potential to validate, refine, or even reclassify the clinical significance of genomic aberrations, thereby improving confidence in the therapeutic decision-making^34,35^.

Dictated by the current clinical practices of genomics-guided cancer medicine, CDSS tools ingest limited patient-specific information, typically only basic descriptors such as tumor type and potentially also information on histological subtype. Contextual factors that may impinge treatment choice, such as comorbidities, performance status (e.g., ECOG), prior lines of treatment, and other physiological parameters related to some common chronic diseases (e.g., hypertension and diabetes), are thus brought into MTB discussions as external information elements. Even more important is the real-world clinical outcome data of CDSS-based recommendations, such as response to therapy or emergence of drug resistance, which the tools are not yet ready to incorporate. Another key and often missed dimension in this regard is that of patient-reported outcomes, or preferences regarding quality of life versus length of life. By interrogating each case in isolation, CDSS tools miss the opportunity to leverage insights from prior decisions that could inform and improve future treatment recommendations. This absence of a feedback loop inevitably prevents the current generation of tools from refining therapy rankings or treatment predictions adaptively, hence overlooking significant contextual patterns that may emerge over time.

## Towards an ecosystem of clinical decision support tools and services

**Fig. 1:**
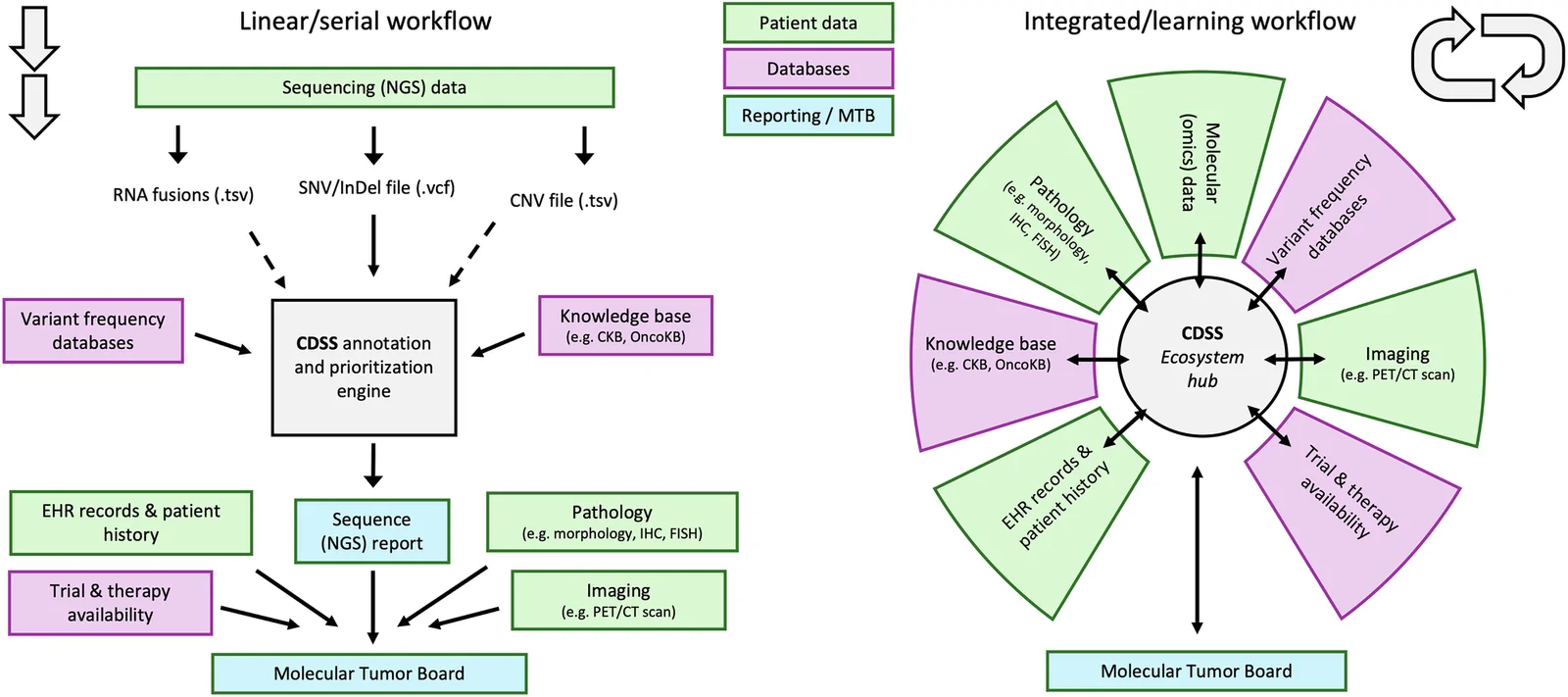
From linear and static interpretation workflows towards integrated and adaptive CDSS ecosystems. A conceptual illustration of the typical architecture of conventional CDSS tools for PCM (left panel, organized as linear/serial workflows) versus an ecosystem-like, modular architecture (right panel), providing a sustainable framework for data integration and adaptive learning. CDSS elements related to input data, external knowledge databases, and output reporting elements are categorized with different colors. CDSS: clinical decision support system, EHR: electronic health record, NGS: next-generation sequencing, IHC: immunohistochemistry, FISH: fluorescence in situ hybridization, PET: positron emission tomography, CT: computed tomography, SNV: single nucleotide variant, InDel: insertion/deletion, CNV: copy-number variant, MTB: molecular tumor board, CKB : Cancer Knowledgebase (Genomenon), OncoKB: Memorial-Sloan Kettering’s precision oncology knowledgebase.

The relatively linear, step-wise operation of current CDSS reporting procedures stands in contrast to the collaborative and multidisciplinary nature of MTBs, in which confidence and consensus in decisions emerge from iterative refinements and support from parallel data interpretations. Considering the growing number of heterogeneous data modalities that may become routinely available per cancer patient, e.g., from single-cell and spatial transcriptomics, additional perspectives and interpretations require facilitation towards the MTB. In light of this situation, the idea of a greater CDSS ecosystem of modular tools and services with dedicated responsibilities may thus represent a more flexible and scalable architectural model than a single unified CDSS platform, and better serve the needs of future MTBs^36^. Here, feedback loops can be created by ensuring that information runs bidirectionally between the governing, core system and its connected services and external knowledgebases (Fig. 1). A pragmatic strategy for the adoption of a CDSS ecosystem, which borrows its architectural principles from microservice software design, is to let it co-exist with the existing CDSS infrastructure, allowing phased roll-out of high-value modules once workflow fit and data flows are stable^8,37^. While feasible, its success hinges upon established interoperability standards in the field (e.g., HL7 FHIR), organizational support and hospital IT infrastructure, and not least iterative refinement from feedback on real-world use cases^38,39^. To support data integration needs and continuous learning in the ecosystem, several emerging approaches that capitalize on generative AI systems, foundation models, and large language models (LLMs) in particular, are highly relevant. Specifically, the significant capacity of LLMs to extract patterns and synthesize knowledge from unstructured text collections, as well as their ability to conduct complex reasoning from heterogeneous input, makes them attractive elements for a CDSS ecosystem.

The potential of multimodal AI models trained on data from large cohorts of cancer patients is considerable, in principle enabling a range of prediction and prioritization opportunities for a single cancer patient^40,41^. Examples include multi-omic actionable variant assessment, tumor subtype identification, and therapy response prediction. Notably, sophisticated therapy response prediction models may cater for context-awareness, incorporating temporal treatment history and comorbidities. Proper uncertainty estimation in multimodal model predictions, as well as inherent data type cross-validation, can furthermore be highly valuable in rare or borderline cases where single modalities exert contradictory or ambiguous signals (e.g., a borderline PD-L1 score). A broader conceptual framework that builds upon multimodal AI models is the digital twin, a virtual representation of the cancer patient that can be used for simulation of treatment response and disease progression^42^. Digital twins are particularly CDSS-relevant due to their support for interactive explorations, providing a causal and mechanistic understanding of tumor growth and response when perturbing the twin with different types of treatments. Substantial work is nevertheless needed to realize the integration of multimodal AI models and digital twins into CDSS tools. A major challenge is to build and leverage robust and unbiased models, requiring large and diverse cohorts with rich clinical and molecular data types, ultimately depending upon cross-institution collaboration efforts. Such efforts are frequently hampered by patient privacy concerns, not least since human genomic data are deemed sensitive according to GDPR. A promising model-building approach that can safeguard patient privacy is that of federated learning, where a model is transported and trained locally on multiple datasets across dataholding sites, such as hospitals. This approach has been performed most significantly with imaging data^43,44^, yet efforts also exist with genomics datasets^45^.

While the future CDSS tools are likely to be influenced by generalist multimodal models, many piloting studies that utilize generative AI have already demonstrated how LLMs can expedite time-consuming parts of data interpretation and decision-making processes towards personalized cancer care^46^. The use of LLMs as reasoning engines rather than mere data extraction tools has emerged as a particularly exciting strategy, where the LLM acts as a clinical assistant or agent that dynamically governs knowledgebases and specialized tools^47^. Critically, LLM-based solutions integrated in CDSS frameworks should, in line with recent ESMO recommendations, be understood as reasoning aids augmenting the MTB discussions, not as autonomous decision-making actors per se^48^. A promising performance of agentic AI-based CDSS architectures has been reported on small test sets^49^. Moreover, an agentic strategy aligns well with the modular CDSS ecosystem proposed here, and where the power of LLM lies in problem-solving more than data extraction procedures per se. When it comes to the latter, LLMs have shown capacity in the extraction, cleaning and structuring of real-world data from health records and pathology reports, and also by facilitating its integration with omics data^50–52^. For more direct MTB-related tasks, the retrieval of treatment recommendations for a given genomic aberration in a specific tumor type has been interrogated in multiple studies, for which general-purpose LLMs (e.g., ChatGPT) have demonstrated their ability to retrieve well-established and approved treatment regimes, albeit on very limited test sets^53,54^. When general-purpose LLMs are supplemented with context and domain-specific knowledge (i.e., retrieval augmentation), through, for instance, relevant medical literature and curated knowledge datasets, the retrieval of relevant recommendations can be substantially improved^55^. Furthermore, matching the characteristics of cancer patients and their tumors to recruiting clinical trials represents another task where LLMs have proven particularly valuable, largely due to the unstructured nature of trial eligibility criteria^56–58^.

Notably, the performance of any LLM-based endeavor toward clinical decision support significantly depends upon the exact query instructions provided to the LLM, a topic known as prompt engineering^59^. A rigorous evaluation of clinician-facing LLM tools is thus critical, a matter being addressed by OpenAI’s recent launch of the HealthBench Professional framework^60^. The focus of that framework is so far related to LLM performance on general clinical reasoning and documentation tasks, which underscores the need for additional benchmarks that assess LLM performance for key precision oncology workflow components, most importantly, variant interpretation towards clinical actionability. Even fine-tuned LLMs, i.e., models supported with domain-specific knowledge, are prone to a considerable level of hallucinations and incoherent reasoning, which highlights the importance of human oversight when such solutions are to be integrated in CDSS ecosystems. The recently proposed recommendations for safe and responsible integration of LLMs with real-world data into CDSS workflows are relevant in this regard, capturing more broadly the ethical, regulatory and legal aspects of deploying AI-based clinical solutions^48,61^.

### Future outlook

The heterogeneous nature of MTB operations and expertise across PCM programs creates different needs and expectations from CDSS tools. While conventional genomics-focused CDSS frameworks represent an essential component for current MTBs, the growing complexity of data modalities becoming available for each cancer patient calls for a larger and more dynamic ecosystem of tools, knowledgebases and services that can enhance the clinical decision-making process. Rather than being presented with the contents of a relatively static NGS report, we envisage a tumor board fueled by a CDSS ecosystem that can dynamically infer clinical actionability evidence through the integration of patient EHR data, multi-omic sequencing results and output from digital pathology assays. An orchestrating AI layer can furthermore aid the reasoning and recommend relevant trials, supporting, while not replacing, the multidisciplinary discussion. Ecosystems capable of continuous learning from clinical outcomes will be essential in transforming conventional rule-based output towards adaptive treatment recommendations. In contrast to institution-tailored CDSS solutions that operate according to a particular PCM program, state-of-the-art ecosystem components (e.g. variant classification engines, trial matching services) can be flexibly combined to better serve the heterogeneous landscape of MTB infrastructures.

There is considerable institutional variation when it comes to regulatory approval for CDSS solutions in MTB infrastructures. Often, a mix of both commercial and academic research-grade tools are employed, in addition to clinical-grade solutions offered by commercial vendors. Even though validated clinical-grade tools build considerable trust in an MTB setting, their internal algorithms and decision logic are proprietary and non-transparent, making it difficult to fully understand and trace their recommendations^24^. The licensing costs can also become prohibitive for smaller institutions or those in low-income settings. The strength of academic solutions is, on the other hand, their transparent nature, with source code and underlying data fully auditable. Most research-grade tools do, however, come without formal clinical validation, partly due to the resource-demanding nature of this procedure. Notably, while regulatory frameworks for AI-based CDSS are still evolving, a modular ecosystem proposed here can allow both research-grade and clinical-grade modules to operate in a hybrid fashion, with clear boundaries between the regulatory and exploratory components. Furthermore, whereas both commercial and open-source tools are faced with sustainability risks, keeping them as modules of an ecosystem rather than monolithic solutions makes the whole system more robust in case any component becomes unsustainable.

Multimodal AI models and digital twins hold considerable promise as strategies for more sophisticated data-driven decision support. Their clinical maturity is, however, challenged by multiple factors, including data sparsity and patient privacy concerns. A fundamental challenge for any AI model in a CDSS ecosystem is rooted in potentially skewed training data - here referring to the common overrepresentation of data from Western/European institutions and populations. Models developed on these data may propagate bias and fail to generalize well for underrepresented ancestries and tumor types. Federated learning frameworks and cross-institutional consortia facilitate model training on more diverse datasets and thus represent part of the solution to this challenge. The incorporation of fairness-aware model evaluation frameworks is also recommended for equity purposes^62^.

Trustworthy and explainable output, as well as compliance with regulatory and ethical standards, will inevitably be necessary for any deployment of AI-based models towards decision support. The performance evaluation of CDSS output, including those from its constituent components, is an intrinsically complex undertaking, primarily since gold standards for CDSS performance are lacking within the field. Ultimately, performance must be measured against the clinical outcomes of patients interrogated and discussed at the molecular tumor board.

## Data Availability

No datasets were generated or analysed during the current study.

## References

[1] Edsjö, A. et al. Precision cancer medicine: concepts, current practice, and future developments. *J. Intern. Med.* **294**, 455–481 (2023). 10.1111/joim.13709 37641393

[2] Edsjö, A. et al. Current and emerging sequencing-based tools for precision cancer medicine. *Mol. Aspects Med.* **96**, 101250 (2024). 10.1016/j.mam.2024.101250 38330674

[3] Kurnit, K. C. et al. Precision oncology decision support: current approaches and strategies for the future. *Clin. Cancer Res.* **24**, 2719–2731 (2018). 10.1158/1078-0432.CCR-17-2494 PMC6004235 29420224

[4] Ciliberto, G. et al. A decalogue of molecular tumor board (MTB) recommendations from the CAN.HEAL Consortium. *Eur. J. Cancer* **222**, 115433 (2025). 10.1016/j.ejca.2025.115433 40253820

[5] Westphalen, C. B. et al. ESMO Precision Oncology Working Group recommendations on the structure and quality indicators for molecular tumour boards in clinical practice. *Ann. Oncol.* **36**, 614–625 (2025). 10.1016/j.annonc.2025.02.009 40194904

[6] Haj Mohammad, S. F. et al. A comprehensive assessment of the existing landscape of personalized cancer medicine in the European Union, on behalf of the PCM4EU consortium. *ESMO Open* **10**, 105872 (2025). 10.1016/j.esmoop.2025.105872 PMC12639420 41197434

[7] Luchini, C., Lawlor, R. T., Milella, M. & Scarpa, A. Molecular tumor boards in clinical practice. *Trends Cancer* **6**, 738–744 (2020). 10.1016/j.trecan.2020.05.008 32517959

[8] Tamborero, D. et al. The Molecular Tumor Board Portal supports clinical decisions and automated reporting for precision oncology. *Nat. Cancer* **3**, 251–261 (2022). 10.1038/s43018-022-00332-x PMC8882467 35221333

[9] Lajmi, N. et al. Challenges and solutions to system-wide use of precision oncology as the standard of care paradigm. *Camb. Prism. Precis. Med.* **2**, e4 (2024). 10.1017/pcm.2024.1 PMC11062796 38699518

[10] Botsis, T. et al. Precision oncology core data model to support clinical genomics decision making. *JCO Clin. Cancer Inform.* **7**, e2200108 (2023). 10.1200/CCI.22.00108 PMC10281442 37040583

[11] Lutz, S. et al. Unveiling the digital evolution of molecular tumor boards. *Target. Oncol.* **20**, 27–43 (2025). 10.1007/s11523-024-01109-1 PMC11762447 39609355

[12] Reisle, C. et al. A platform for oncogenomic reporting and interpretation. *Nat. Commun.* **13**, 756 (2022). 10.1038/s41467-022-28348-y PMC8828759 35140225

[13] Tamborero, D. et al. Cancer genome interpreter annotates the biological and clinical relevance of tumor alterations. *Genome Med.* **10**, 25 (2018). 10.1186/s13073-018-0531-8 PMC5875005 29592813

[14] Reardon, B. et al. Integrating molecular profiles into clinical frameworks through the Molecular Oncology Almanac to prospectively guide precision oncology. *Nat. Cancer.* **2**, 1102–1112 (2021). 10.1038/s43018-021-00243-3 PMC9082009 35121878

[15] Nakken, S. et al. Personal cancer genome reporter: variant interpretation report for precision oncology. *Bioinformatics* **34**, 1778–1780 (2018). 10.1093/bioinformatics/btx817 PMC5946881 29272339

[16] Patterson, S. E., Statz, C. M., Yin, T. & Mockus, S. M. Utility of the JAX Clinical Knowledgebase in capture and assessment of complex genomic cancer data. *NPJ Precis. Oncol.* **3**, 2 (2019). 10.1038/s41698-018-0073-y PMC6333807 30675517

[17] Chakravarty, D. et al. OncoKB: a precision oncology knowledge base. *JCO Precis. Oncol.* **2017** (2017). 10.1200/PO.17.00011 PMC5586540 28890946

[18] Griffith, M. et al. CIViC is a community knowledgebase for expert crowdsourcing the clinical interpretation of variants in cancer. *Nat. Genet.* **49**, 170–174 (2017). 10.1038/ng.3774 PMC5367263 28138153

[19] Frazer, J. et al. Disease variant prediction with deep generative models of evolutionary data. *Nature* **599**, 91–95 (2021). 10.1038/s41586-021-04043-8 34707284

[20] Cheng, J. et al. Accurate proteome-wide missense variant effect prediction with AlphaMissense. *Science* **381**, eadg7492 (2023). 10.1126/science.adg7492 37733863

[21] Horak, P. et al. Standards for the classification of pathogenicity of somatic variants in cancer (oncogenicity): Joint recommendations of Clinical Genome Resource (ClinGen), Cancer Genomics Consortium (CGC), and Variant Interpretation for Cancer Consortium (VICC). *Genet. Med*. 10.1016/j.gim.2022.01.001 (2022). PMC9081216 35101336

[22] Li, M. M. et al. Assessments of somatic variant classification using the Association for Molecular Pathology/American Society of Clinical Oncology/College of American Pathologists guidelines: a report from the Association for Molecular Pathology. *J. Mol. Diagn.* **25**, 69–86 (2023). 10.1016/j.jmoldx.2022.11.002 36503149

[23] Mateo, J. et al. A framework to rank genomic alterations as targets for cancer precision medicine: the ESMO scale for clinical actionability of molecular targets (ESCAT). *Ann. Oncol.* **29**, 1895–1902 (2018). 10.1093/annonc/mdy263 PMC6158764 30137196

[24] Perakis, S. O. et al. Comparison of three commercial decision support platforms for matching of next-generation sequencing results with therapies in patients with cancer. *ESMO Open* **5**, e000872 (2020). 10.1136/esmoopen-2020-000872 PMC7513637 32967919

[25] Budczies, J. et al. Tumour mutational burden: clinical utility, challenges and emerging improvements. *Nat. Rev. Clin. Oncol.* **21**, 725–742 (2024). 10.1038/s41571-024-00932-9 39192001

[26] Andrews, H. S. et al. Analysis of 20 independently performed assays to measure Homologous recombination deficiency (HRD) in ovarian cancer: findings from the Friends’ HRD harmonization project. *JCO Oncol. Adv*. 10.1200/oa-24-00042 (2024).

[27] Vaske, O. M. et al. Comparative tumor RNA sequencing analysis for difficult-to-treat pediatric and young adult patients with cancer. *JAMA Netw. Open* **2**, e1913968 (2019). 10.1001/jamanetworkopen.2019.13968 PMC6822083 31651965

[28] Pleasance, E. et al. Whole genome and transcriptome analysis enhances precision cancer treatment options. *Ann. Oncol*. 10.1016/j.annonc.2022.05.522 (2022). 35691590

[29] Ellrott, K. et al. Classification of non-TCGA cancer samples to TCGA molecular subtypes using compact feature sets. *Cancer Cell* **43**, 195–212.e11 (2025). 10.1016/j.ccell.2024.12.002 PMC11949768 39753139

[30] Nakken, S. et al. Cancer predisposition sequencing reporter (CPSR): a flexible variant report engine for high-throughput germline screening in cancer. *Int. J. Cancer* **149**, 1955–1960 (2021). 10.1002/ijc.33749 34310709

[31] Farmer, H. et al. Targeting the DNA repair defect in BRCA mutant cells as a therapeutic strategy. *Nature* **434**, 917–921 (2005). 10.1038/nature03445 15829967

[32] Shin, H.-T. et al. Prevalence and detection of low-allele-fraction variants in clinical cancer samples. *Nat. Commun.* **8**, 1377 (2017). 10.1038/s41467-017-01470-y PMC5680209 29123093

[33] Li, Y. et al. Pan-cancer proteogenomics connects oncogenic drivers to functional states. *Cell* **186**, 3921–3944.e25 (2023). 10.1016/j.cell.2023.07.014 37582357

[34] Bollas, A., Gaither, J., Schieffer, K. M., White, P. & Mardis, E. R. Variant calling from RNA-Seq data reveals allele-specific differential expression of pathogenic cancer variants. *Commun. Med.* **5**, 202 (2025). 10.1038/s43856-025-00901-y PMC12119874 40437029

[35] Li, D. et al. Augmenting precision medicine via targeted RNA-Seq detection of expressed mutations. *NPJ Precis. Oncol.* **9**, 182 (2025). 10.1038/s41698-025-00993-8 PMC12166063 40514442

[36] Walsh, S. et al. Decision support systems in oncology. *JCO Clin. Cancer Inform.* **3**, 1–9 (2019). 10.1200/CCI.18.00001 PMC6873918 30730766

[37] Warner, J. L. et al. SMART Cancer Navigator: a framework for implementing ASCO workshop recommendations to enable precision cancer medicine. *JCO Precis. Oncol.* **2018**, 1–14 (2018). 10.1200/PO.17.00292 PMC6141039 30238071

[38] Orenstein, E. W. et al. Towards a maturity model for clinical decision support operations. *Appl. Clin. Inform.* **10**, 810–819 (2019). 10.1055/s-0039-1697905 PMC6821535 31667818

[39] Hill, A., Morrissey, D. & Marsh, W. What characteristics of clinical decision support system implementations lead to adoption for regular use? A scoping review. *BMJ Health Care Inform.* **31**, e101046 (2024). 10.1136/bmjhci-2024-101046 PMC11344512 39181544

[40] Lipkova, J. et al. Artificial intelligence for multimodal data integration in oncology. *Cancer Cell* **40**, 1095–1110 (2022). 10.1016/j.ccell.2022.09.012 PMC10655164 36220072

[41] Keyl, J. et al. Decoding pan-cancer treatment outcomes using multimodal real-world data and explainable artificial intelligence. *Nat. Cancer* **6**, 307–322 (2025). 10.1038/s43018-024-00891-1 PMC11864985 39885364

[42] Hernandez-Boussard, T. et al. Digital twins for predictive oncology will be a paradigm shift for precision cancer care. *Nat. Med.* **27**, 2065–2066 (2021). 10.1038/s41591-021-01558-5 PMC9097784 34824458

[43] Ogier du Terrail, J. et al. Federated learning for predicting histological response to neoadjuvant chemotherapy in triple-negative breast cancer. *Nat. Med.* **29**, 135–146 (2023). 10.1038/s41591-022-02155-w 36658418

[44] Liu, Y. et al. Predicting treatment response in multicenter non-small cell lung cancer patients based on federated learning. *BMC Cancer* **24**, 688 (2024). 10.1186/s12885-024-12456-7 PMC11155008 38840081

[45] Wang, Q., He, M., Guo, L. & Chai, H. AFEI: adaptive optimized vertical federated learning for heterogeneous multi-omics data integration. *Brief. Bioinform*. **24**, bbad269 (2023). 10.1093/bib/bbad269 37497720

[46] Derbal, Y. Reimagining cancer treatments in the era of generative AI. *Digit. Health* **11**, 20552076251394631 (2025). 10.1177/20552076251394631 PMC12583873 41195370

[47] Karunanayake, N. Next-generation agentic AI for transforming healthcare. *Inform. Health* **2**, 73–83 (2025).

[48] Wong, E. Y. T. et al. ESMO guidance on the use of large language models in clinical practice (ELCAP). *Ann. Oncol.* **36**, 1447–1457 (2025). 10.1016/j.annonc.2025.09.001 41111032

[49] Ferber, D. et al. Development and validation of an autonomous artificial intelligence agent for clinical decision-making in oncology. *Nat. Cancer*10.1038/s43018-025-00991-6 (2025). PMC12380607 40481323

[50] Truhn, D. et al. Extracting structured information from unstructured histopathology reports using generative pre-trained transformer 4 (GPT-4). *J. Pathol.* **262**, 310–319 (2024). 10.1002/path.6232 38098169

[51] Truhn, D., Eckardt, J.-N., Ferber, D. & Kather, J. N. Large language models and multimodal foundation models for precision oncology. *NPJ Precis. Oncol.* **8**, 72 (2024). 10.1038/s41698-024-00573-2 PMC10959931 38519519

[52] Wiest, I. C. et al. A software pipeline for medical information extraction with large language models, open source and suitable for oncology. *NPJ Precis. Oncol.* **9**, 313 (2025). 10.1038/s41698-025-01103-4 PMC12443949 40962856

[53] Benary, M. et al. Leveraging large language models for decision support in personalized oncology. *JAMA Netw. Open* **6**, e2343689 (2023). 10.1001/jamanetworkopen.2023.43689 PMC10656647 37976064

[54] Burnette, H. et al. Chatbot assistance in precision oncology treatment decision-making. *Oncologist* **30**, oyaf316 (2025). 10.1093/oncolo/oyaf316 PMC12517745 41014149

[55] Lammert, J. et al. Expert-guided large language models for clinical decision support in precision oncology. *JCO Precis. Oncol.* **8**, e2400478 (2024). 10.1200/PO-24-00478 39475661

[56] Abdallah, M. et al. TrialMatchAI: an end-to-end AI-powered clinical trial recommendation system to streamline patient-to-trial matching. *Nat. Commun.* **17,** 4472 (2026). 10.1038/s41467-026-70509-w PMC13187043 41876500

[57] Jin, Q. et al. Matching patients to clinical trials with large language models. *Nat. Commun.* **15**, 9074 (2024). 10.1038/s41467-024-53081-z PMC11574183 39557832

[58] Schulman, A. et al. In *Applied Artificial Intelligence for Drug Discovery* (ed. Lavecchia, A.), Ch. 24 (Springer, 2026).

[59] Carl, N. et al. Large language model use in clinical oncology. *NPJ Precis. Oncol.* **8**, 240 (2024). 10.1038/s41698-024-00733-4 PMC11499929 39443582

[60] Hicks, R. S. et al. HealthBench professional: evaluating large language models on real clinician chats. Preprint at arXiv 10.48550/arXiv.2604.27470 (2026).

[61] Mathes, S. et al. Collaborative framework on responsible AI in LLM-driven CDSS for precision oncology leveraging real-world patient data. *NPJ Precis. Oncol*. **10**, 15 (2025). 10.1038/s41698-025-01180-5 PMC12796327 41345244

[62] Dankwa-Mullan, I. & Weeraratne, D. Artificial intelligence and machine learning technologies in cancer care: Addressing disparities, bias, and data diversity. *Cancer Discov.* **12**, 1423–1427 (2022). 10.1158/2159-8290.CD-22-0373 PMC9662931 35652218

